# Advancements and Challenges in Peptide-Based Cancer Vaccination: A Multidisciplinary Perspective

**DOI:** 10.3390/vaccines12080950

**Published:** 2024-08-22

**Authors:** Dequan Liu, Lei Liu, Xinghan Li, Shijin Wang, Guangzhen Wu, Xiangyu Che

**Affiliations:** 1 Department of Urology, The First Affiliated Hospital of Dalian Medical University, Dalian 116011, China; liudq@dmu.edu.cn (D.L.); liuleidmu1989@163.com (L.L.); wangsj01@dmu.edu.cn (S.W.); 2 Department of Stomatology, General Hospital of Northern Theater Command, Shenyang 110016, China; lxh950601@163.com

**Keywords:** peptide-based cancer vaccines, mechanism of action, combination therapy, medication administration, adjuvants, delivery systems

## Abstract

With the continuous advancements in tumor immunotherapy, researchers are actively exploring new treatment methods. Peptide therapeutic cancer vaccines have garnered significant attention for their potential in improving patient outcomes. Despite its potential, only a single peptide-based cancer vaccine has been approved by the U.S. Food and Drug Administration (FDA). A comprehensive understanding of the underlying mechanisms and current development status is crucial for advancing these vaccines. This review provides an in-depth analysis of the production principles and therapeutic mechanisms of peptide-based cancer vaccines, highlights the commonly used peptide-based cancer vaccines, and examines the synergistic effects of combining these vaccines with immunotherapy, targeted therapy, radiotherapy, and chemotherapy. While some studies have yielded suboptimal results, the potential of combination therapies remains substantial. Additionally, we addressed the management and adverse events associated with peptide-based cancer vaccines, noting their relatively higher safety profile compared to traditional radiotherapy and chemotherapy. Lastly, we also discussed the roles of adjuvants and targeted delivery systems in enhancing vaccine efficacy. In conclusion, this review comprehensively outlines the current landscape of peptide-based cancer vaccination and underscores its potential as a pivotal immunotherapy approach.

## 1. Introduction

The concept of immunotherapy dates back to the late 19th century when William Coley, a New York surgeon, observed that infections in cancer patients could occasionally lead to tumor regression [1]. This observation led to the development of Coley’s toxins, who had been exploiting streptococcus-mediated tumor rejection, a form of early immunotherapy [2,3]. The identification of tumor antigens in the late 20th century was a pivotal moment for cancer vaccines [4,5]. Tumor antigens are proteins or peptides presented on the surface of cancer cells that can be recognized by the immune system [6,7,8]. This discovery led to the idea that vaccines could be developed to stimulate the immune system to recognize and attack tumors based on these antigens [6,7,8]. Peptide-based cancer vaccines are designed to specifically target these tumor antigens, aiming to elicit a strong and specific immune response against cancer cells [9].

Peptide-based cancer vaccines have emerged as a promising strategy in cancer immunotherapy [10,11]. Peptide-based cancer vaccines work by inducing an immune response against specific tumor-associated antigens (TAAs) derived from mutated or overexpressed proteins in cancer cells [9,11]. These vaccines are designed to be recognized by the immune system, particularly cytotoxic T lymphocytes (CTLs), which can then target and kill cancer cells presenting these antigens [12]. The first peptide-based cancer vaccines were developed in the 1990s, with initial trials focusing on melanoma, namely melanoma antigen gene-1 (MAGE-1)-derived peptide-based cancer vaccines [13,14,15]. Specifically, the researchers found that human melanoma cells can process the MAGE-1 gene product and present the processed nonapeptide EADPTGHSY on their human leukocyte antigen (HLA)-A1 molecules as a determinant for CTLs [15]. Immunizing melanoma patients, whose tumor cells express the MAGE-1 gene and who are HLA-A1+, with cultured autologous antigen-presenting cells pulsed with the synthetic nonapeptide-induced autologous melanoma-reactive and nonapeptide-specific CTLs in situ at the immunization site and at distant metastatic sites demonstrated the potential of peptide vaccines in cancer immunotherapy [13,15]. Early clinical trials explored the immunogenicity and therapeutic potential of peptides derived from melanoma-associated antigens [16]. These studies laid the groundwork for understanding how peptide vaccines could be optimized for better immune response.

Despite facing challenges such as variability in patient responses and the need for enhanced targeting, ongoing research is dedicated to overcoming these obstacles through novel adjuvants, combination therapies, and personalized vaccine strategies [10,17]. Combining peptide vaccines with immune checkpoint inhibitors, such as anti-PD-1/PD-L1 and anti-cytotoxic T-lymphocyte-associated protein 4 (CTLA-4) antibodies, has shown promising results by counteracting the immunosuppressive tumor microenvironment [18]. Additionally, combining peptide vaccines with conventional therapies like chemotherapy and radiation has been explored to modulate the tumor microenvironment and enhance the efficacy of immunotherapy [19]. Strategies such as using neoantigens and incorporating multi-epitope peptides to target multiple pathways aim to overcome tumor heterogeneity [17]. Despite the challenges and limited clinical successes, peptide-based therapeutic cancer vaccines remain a hopeful avenue in cancer treatment. Sipuleucel-T (Provenge) was approved by the FDA in April 2010, developed by Dendreon Corporation, and is designed to stimulate an immune response against prostate cancer using the patient’s dendritic cells loaded and activated with a fusion protein [20]. Sipuleucel-T, an autologous active cellular immunotherapy, demonstrated efficacy in reducing the risk of death among men with metastatic castration-resistant prostate cancer [20]. The phase 3 trial revealed a 22% relative reduction in the risk of death for patients treated with Sipuleucel-T compared to a placebo, translating to a 4.1-month improvement in median survival [20]. Although Sipuleucel-T is not a traditional peptide vaccine, it utilizes a recombinant fusion protein that includes prostatic acid phosphatase, a prostate antigen, linked to an immune cell activator (GM-CSF) [20]. This brings hope to relevant researchers. A better understanding of the immune response, optimization of vaccine formulations, and strategic combinations with other therapies are crucial for developing the next generation of these vaccines [10]. In this review, we examine the production processes and mechanisms of action of peptide tumor vaccines and provide an overview of various peptide-based therapeutic cancer vaccines. Additionally, we discuss the opportunities and challenges encountered in their clinical application.

## 2. The Current Status of Peptide Vaccine Development: Promising Potential Amidst Challenges

The general rate of success of cancer peptide vaccines, in terms of overall survival (OS) and progression-free survival (PFS), has been variable and generally modest [21,22]. For instance, in a phase 3 randomized trial, patients with advanced melanoma treated with the gp100 peptide vaccine and interleukin-2 showed a significantly higher overall clinical response rate (16% vs. 6%) and longer PFS (2.2 vs. 1.6 months) compared to those receiving interleukin-2 alone [21]. The study, involving 185 patients, demonstrated a median OS of 17.8 months for the vaccine-interleukin-2 group compared to 11.1 months for the interleukin-2-only group [21]. Meanwhile, in a recent placebo-controlled trial, 53 participants received the MUC1 peptide vaccine, while 50 received a placebo to prevent recurrent colorectal adenoma [22]. Thirteen of fifty-two (25%) vaccine recipients exhibited a significant immune response, and among these immune responders, a 38% absolute reduction in adenoma recurrence was observed compared to the placebo group [22]. Despite these outcomes, peptide vaccines generally exhibit less impressive results compared to other immunotherapies [23,24,25]. For instance, immune checkpoint inhibitors have demonstrated higher response rates in clinical trials for solid tumors, including non-small cell lung cancer, renal cell carcinoma, and melanoma [26,27,28].

In summary, due to the unsatisfactory performance of peptide vaccines in clinical trials and various inherent challenges and limitations, the FDA has approved only one peptide vaccine for cancer treatment: Sipuleucel-T (Provenge) [29]. First and foremost, the primary limitation of peptide vaccines is their low immunogenicity, which means they typically do not elicit strong immune responses independently [17]. This is partly due to the short length of the peptides, which may not be sufficient to activate robust T-cell responses [17]. Enhancing immunogenicity through potent adjuvants, longer peptides, and peptide cocktails can boost immune responses [17,30]. Another challenge is that peptide vaccines are usually designed to bind to specific HLA (human leukocyte antigen) molecules [31]. If only a single target peptide is used for vaccination, immune escape may occur [32]. Additionally, because HLA molecules vary greatly between individuals, a peptide vaccine effective for one person may not be effective for another, limiting its universal applicability [33]. To address this, current strategies include designing vaccines that contain multiple epitopes capable of binding to different HLA molecules, thereby increasing the likelihood of benefiting a broader patient population [32,34]. Additionally, developing personalized vaccines tailored to the HLA characteristics of individual patients can enhance the effectiveness of peptide vaccines [35,36].

## 3. Mechanism of Action of Peptide Cancer Vaccines

**Figure 1:**
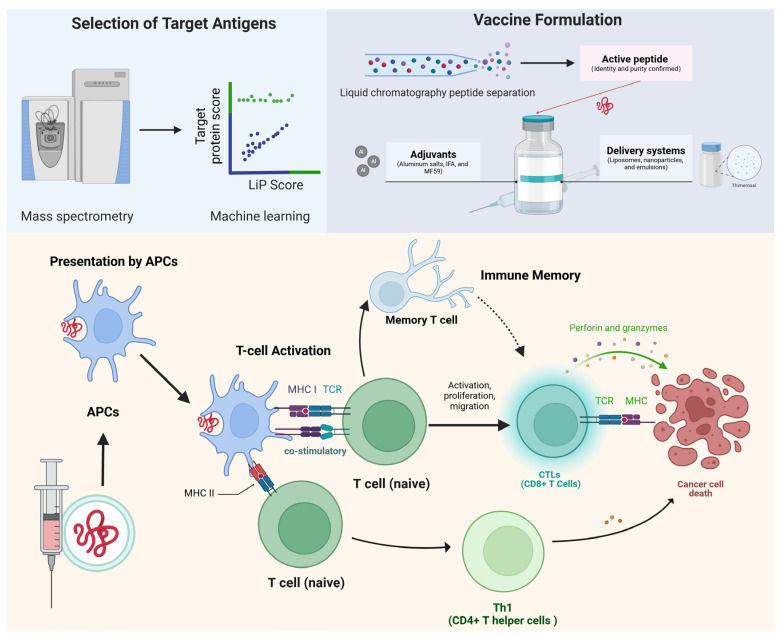
Preparation and mechanism of peptide cancer vaccine. Peptide-based cancer vaccines stimulate the immune system to target and destroy cancer cells by using synthesized peptides that represent specific antigens overexpressed or unique to cancer cells, which are formulated with adjuvants and presented by APCs to T cells, thereby activating them to attack cancer cells and potentially forming memory T cells to prevent recurrence. APCs: antigen-presenting cells; TCR: T-cell receptors; MHC: major histocompatibility complex; CTLs: cytotoxic T lymphocytes. Created with https://www.biorender.com, accessed on 1 June 2024.

Peptide-based cancer vaccines work by stimulating the immune system to target and destroy cancer cells [9]. Researchers select specific antigens that are overexpressed or unique to cancer cells, synthesize peptides that represent these antigens, and then formulate these peptides with adjuvants to enhance the immune response [9]. Once administered, antigen-presenting cells (APCs) present these peptides to T cells, activating them to attack cancer cells and potentially form memory T cells to prevent recurrence [10,37]. The mechanism of action of peptide-based cancer vaccines involves a series of steps designed to elicit an immune response against cancer cells (Figure 1).

### 3.1. Selection of Target Antigens

Key considerations in designing peptide vaccines include optimizing peptide length, selecting highly immunogenic epitopes, and balancing B-cell and T-cell responses [38,39]. Overcoming the immunosuppressive tumor microenvironment (TME) is crucial for the efficacy of peptide vaccines [40]. Potent adjuvants are critical for enhancing the immunogenicity of peptide vaccines, which can stimulate innate immune responses, leading to the activation of dendritic cells and the subsequent priming of T cells [41,42]. Meanwhile, peptide vaccines employ various types of peptides, including linear peptides, modified peptides, and peptides of different lengths, to effectively target cancer cells [43,44,45]. Linear peptides are straightforward sequences designed to stimulate specific immune responses, while modified peptides undergo chemical alterations to enhance stability, binding affinity, and immunogenicity [43,44,45]. Short peptides (8–10 amino acids) typically target T-cell epitopes to elicit cytotoxic responses, whereas long peptides (20–30 amino acids) can contain multiple epitopes to stimulate both CD4+ and CD8+ T cells [46,47,48]. Multi-epitope peptides and neoantigen peptides tailored to tumor-specific mutations provide broader and more personalized immune responses, respectively [39,48]. However, there is the potential for autoimmunity and immune escape, especially for TAAs, as the immune system may attack normal cells expressing these antigens [41,49]. In contrast, tumor-specific antigens (TSAs) that specifically target cancer cells pose less risk [49]. Tumor immune escape through antigen mutation or downregulation, as well as tumor heterogeneity, complicate vaccine efficacy for both TAA and TSA vaccines [9]. Developing vaccines against neoantigens requires a personalized approach, which is more complex and costly but offers greater specificity and a reduced risk of autoimmunity [50,51]. Additionally, direct tumor targeting through intratumoral injection can focus the immune response within the TME [50,51]. These strategies are essential for developing effective peptide vaccines capable of overcoming the TME and eliciting a strong, targeted immune response against cancer cells.

Advances in high-throughput sequencing and mass spectrometry have led to the identification of numerous TAAs and TSAs [52,53,54,55]. TAAs are proteins or peptides overexpressed in cancer cells compared to normal cells, including examples like carcinoembryonic antigen (CEA) and prostate-specific antigen (PSA) [56,57]. TSAs, arising from mutations, are unique to cancer cells, exemplified by neoantigens from Kirsten rat sarcoma viral oncogene homolog (KRAS) or p53 mutations [58,59]. Bioinformatics tools have significantly improved the prediction of immunogenic epitopes and their binding to major histocompatibility complex (MHC) molecules, facilitating the selection of peptides capable of eliciting robust immune responses [60,61]. For example, Nielsen and Andreatta discussed the NetMHCpan-3.0 tool, which enhances peptide-binding prediction to MHC class I molecules, while Jespersen et al. highlighted the BepiPred-2.0 tool for improved B-cell epitope prediction [62,63]. Additionally, advanced genomic and proteomic technologies, like next-generation sequencing and mass spectrometry, play crucial roles in identifying potential antigens and validating their immunogenicity [64,65]. Robbins et al. demonstrated the use of next-generation sequencing to identify mutated antigens recognized by tumor-reactive T cells [66]. Enhanced peptide synthesis and validation processes, including MHC binding assays and T-cell activation tests, have increased the reliability of candidate peptides [67,68]. Ongoing innovations in neoantigen discovery, supported by advancements in artificial intelligence and machine learning, continue to enhance the efficacy and applicability of peptide-based cancer vaccines [17,69]. Peptides representing these antigens are synthetically optimized for stability, immune recognition, and MHC molecule binding, aiming to induce a robust and specific immune response against cancer cells while minimizing autoimmunity or tolerance issues [9,10,37] (Figure 1). Common tumor antigens and their respective characteristics in recent years are shown in Table 1.

### 3.2. Vaccine Formulation

The peptide synthesis, typically through solid-phase peptide synthesis, efficiently assembles the amino acid sequence [87]. The peptides then undergo purification, often via high-performance liquid chromatography (HPLC), to remove impurities, and are characterized to confirm their identity and purity using methods like mass spectrometry and analytical HPLC [88]. To enhance the immune response, the peptides are formulated with adjuvants [30,89]. Adjuvants are chosen for their ability to enhance the immune response, including aluminum salts, incomplete Freund’s adjuvant (IFA), and MF59, which are selected to enhance the immune response by stimulating the innate immune system and facilitating antigen presentation [30,89]. Delivery systems such as liposomes, nanoparticles, and emulsions are utilized to protect peptides from degradation, ensure targeted delivery, and facilitate uptake by antigen-presenting cells [9,90,91,92]. The formulation must also address peptide stability, optimizing conditions to prevent degradation. Factors affecting immunogenicity include the mode of administration, dose, and dosing schedule [30,93]. The final product, formulated as a liquid for injection or lyophilized for reconstitution, is typically administered via subcutaneous, intradermal, or intramuscular injection, depending on the specific vaccine design [91,92]. Additionally, formulations are developed with an eye toward regulatory standards and manufacturability, ensuring the vaccine can be produced at scale and meet safety, efficacy, and quality benchmarks [94,95] (Figure 1).

### 3.3. Presentation and T-Cell Activation in Peptide-Based Cancer Vaccines

The presentation of antigens by APCs such as dendritic cells and macrophages is essential for initiating immune responses against cancer through peptide-based vaccines [96,97]. Internalized peptides are processed into smaller fragments and presented on MHC class I or class II molecules on the surface of APCs, which then migrate to lymphoid organs to present these complexes to T cells, thereby initiating adaptive immune responses [98,99,100,101]. T-cell activation involves the interaction between the T-cell receptor (TCR) on the T cell and the peptide–MHC complex on the APC, along with necessary co-stimulatory signals [102,103]. CD8+ T cells differentiate into CTLs that can directly kill cancer cells, while CD4+ T cells secrete cytokines to enhance immune responses [98,100]. Immunological memory, mediated by central and effector memory T cells, enables the immune system to rapidly recognize and combat previously encountered cancer cells (Figure 1).

### 3.4. Enhancing CTL Response Induction

Cytotoxic T-lymphocyte (CTL) responses are crucial for anti-tumor immunity within peptide-based cancer vaccines, but their induction requires precise antigen delivery mechanisms [104]. Dendritic cells (DCs) are pivotal, capturing and cross-presenting tumor antigens on MHC class I molecules to activate CTLs [35]. Enhancing CTL responses involves the use of adjuvants and cytokines to boost DC function [41,105,106]. For instance, in a phase I clinical trial, a peptide vaccination emulsified in IFA successfully elicited leukemia-associated antigen-specific cytotoxic CD8+ T-cell responses in patients with chronic lymphocytic leukemia (CLL) [107]. Meanwhile, adjuvants for peptide vaccines are essential for eliciting robust anti-tumor CD4+ T-cell responses [108]. For instance, the combination of synthetic peptides with Toll-like receptor (TLR) agonists and OX40/CD40 co-stimulation has been shown to produce significant anti-tumor effects in a mouse model of malignant melanoma [108]. Additionally, immune checkpoint inhibitors prevent negative regulatory signals, reinvigorating CTLs [109]. Combination therapies enhance CTL efficacy by increasing tumor antigen release and susceptibility of tumor cells to immune attack [9]. Advanced delivery systems, such as nanoparticles, improve antigen presentation and CTL induction [35]. Together, these strategies aim to enhance the anti-tumor immunity of peptide tumor vaccines.

### 3.5. Cold Chain Transport

Maintaining an effective cold chain is critical for the stability and effectiveness of peptide tumor vaccines, particularly in resource-limited settings where inconsistencies during transportation and storage can compromise efficacy [110]. Regulatory oversight and the use of electronic and chemical monitoring devices are essential to mitigate risk and ensure a robust cold chain [110]. These regions face significant challenges, including unreliable electricity, limited refrigeration, difficult terrain, extreme climates, and funding constraints [111,112]. Addressing these challenges requires strategies such as developing thermostable vaccine formulations, investing in health infrastructure, fostering partnerships, and providing training and capacity building to support reliable cold chain systems [111,112]. For instance, in Tunisia, continuous temperature monitoring combined with other technological interventions significantly reduced the prevalence of accidental vaccine freezing [113]. The incidence of freeze alarms at health center levels dropped by 40%, and the risk of freezing during transport decreased from 13.8% to 1.7%, demonstrating the effectiveness of temperature monitoring in protecting vaccine potency [113]. Additionally, peptides in lyophilized form are chemically stable even at ambient temperatures, eliminating the need for continuous cold chain storage and transportation required for traditional vaccines [114]. These findings underscore the importance of developing robust cold chain transportation systems for peptide tumor vaccines.

## 4. Current Peptide-Based Cancer Vaccines

Common peptide-based cancer vaccines currently in clinical practice and trials include GV1001 targeting hTERT in pancreatic and NSCLC (non-small-cell lung cancer), IMA901 for renal cell carcinoma, HER2/neu peptide vaccines like NeuVax and GP2 for breast cancer, NY-ESO-1 vaccines for melanoma and ovarian cancer, WT1 vaccines for leukemia and mesothelioma, and MAGE-A3 vaccines for melanoma and NSCLC [115,116,117,118,119,120,121,122,123]. Recently, a novel intranasal peptide vaccine significantly inhibited NSCLC in an inducible mutant KRAS-mouse lung tumor model [124]. The immunized animals showed decreased CD4 + FoxP3 + T cells, increased interferon (IFN)-γ and IL-17a levels, enhanced KRAS-specific Th1 and Th17 responses, and significantly reduced tumor incidence compared to controls [124]. More information about peptide-based cancer vaccines is shown in Table 2. Due to tumor heterogeneity and immune escape mechanisms, peptide vaccines are rarely used as monotherapy [9]. Tumor heterogeneity, including intratumoral and intertumoral variability, results in diverse subpopulations of cancer cells with distinct genetic and phenotypic characteristics, making it difficult for a single vaccine to target all tumor cells effectively [40,125,126]. Immune escape mechanisms, such as antigen loss, altered antigen presentation, creation of an immunosuppressive microenvironment, and upregulation of immune checkpoint molecules, further diminish the efficacy of vaccines [40,41]. These factors lead to limited efficacy, incomplete tumor eradication, and potential relapse when vaccines are used alone [38]. The current focus is on identifying suitable combination therapies, as well as developing effective adjuvants and delivery systems [9].

## 5. Peptide-Based Cancer Vaccines Combination Therapy: A Promising Approach

**Figure 2:**
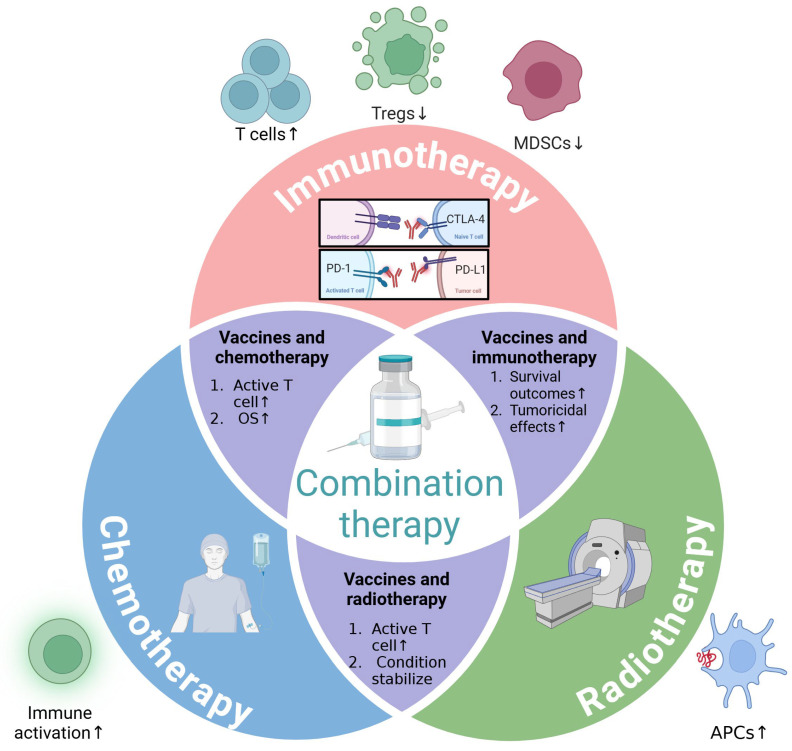
Peptide-based cancer vaccines combined with immunotherapy, radiotherapy, and chemotherapy. Combining peptide-based cancer vaccines with immunotherapy drugs enhances the priming and expansion of T cells while reducing immunosuppressive cells such as Tregs and MDSCs, leading to improved tumoricidal efficacy and survival outcomes in clinical trials. Additionally, combining these vaccines with chemotherapy has shown to strengthen anti-tumor immune responses, boost T cell activation, and improve OS in patients, while their combination with radiation therapy induces immunogenic cell death, promotes the release and uptake of TAAs by APCs, enhances T cell activation, and stabilizes disease progression in clinical trials. APCs: antigen-presenting cells; Tregs: regulatory T cells; MDSCs: myeloid-derived suppressor cells; OS: overall survival. Created with https://www.biorender.com, accessed on 1 June 2024.

Combination therapy involving peptide-based cancer vaccines leverages the synergistic effects of multiple treatment modalities to enhance the immune response against tumors [17,19]. By combining peptide-based cancer vaccines with immune checkpoint inhibitors, the immune system’s ability to recognize and destroy cancer cells is significantly improved [18,148,149,150]. This approach helps to overcome the immunosuppressive environment often created by tumors, thereby boosting the efficacy of the vaccines [148,151]. Additionally, integrating peptide-based cancer vaccines with conventional therapies like chemotherapy and radiation can modulate the tumor microenvironment, making it more conducive to an immune attack [152,153,154,155,156]. Ongoing research and clinical trials continue to explore and refine these combination therapies, aiming to establish them as standard treatment options in oncology [153] (Figure 2).

### 5.1. Combination Therapy with Targeted Therapy and Immunotherapy

There are few studies on the combination of peptide-based cancer vaccines and targeted therapies, and experiments have shown that the effect of this combination therapy is modest [117]. In 2016, in a multicenter, open-label, randomized, controlled phase 3 trial, Rini et al. evaluated the efficacy of the IMA901 peptide-based cancer vaccine plus sunitinib versus sunitinib alone as first-line therapy for advanced or metastatic renal cell carcinoma (IMPRINT) [117]. The study found that the combination of IMA901 and sunitinib did not significantly improve OS compared to sunitinib alone [117]. While the vaccine induced strong immune responses, the median OS was similar between both groups, indicating that further development of IMA901 requires enhancement of its immune response magnitude [117].

Compared with the combination with targeted therapy, researchers have conducted more in-depth research on the combination of peptide-based cancer vaccines and immunotherapy, and the progress achieved has been more significant [9]. By combining peptide-based cancer vaccines with immunotherapy drugs, the vaccines can better prime and expand T cells, leading to a more potent and sustained anti-tumor effect [42,157,158]. Immunotherapy can modulate the tumor microenvironment, reducing immunosuppressive cells like regulatory T cells (Tregs) and myeloid-derived suppressor cells (MDSCs), thus enhancing the function and infiltration of effector T cells [158,159,160]. This synergy is in line with the principles of personalized medicine and optimizing treatment efficacy [9] (Figure 2).

As early as the late 1990s, Disis et al. demonstrated the generation of immunity to the HER-2/neu oncogenic protein in patients with breast and ovarian cancer using a peptide-based vaccine [149]. The vaccine included peptides derived from the HER-2/neu extracellular and intracellular domains, mixed with granulocyte macrophage colony-stimulating factor as an adjuvant, to elicit a CD4 T-helper-specific immune response [149]. All patients immunized with HER-2/neu peptides developed peptide-specific T-cell responses, with the majority also showing protein-specific responses and potential for tumor site migration [149]. Subsequently, more clinical trials verified the superiority of the combination of immunotherapy drugs and peptide-based cancer vaccines. In 2010, in a phase 3 study (NCT00094653) conducted by Hodi et al., ipilimumab, an anti-CTLA-4 antibody, was evaluated for its efficacy in improving OS in patients with metastatic melanoma [150]. The study involved 676 patients who were randomly assigned to receive either ipilimumab plus the gp100 peptide vaccine, ipilimumab alone, or the gp100 peptide vaccine alone [150]. The results demonstrated that the median OS was significantly longer in the group receiving the combination of ipilimumab and the gp100 peptide vaccine (10.0 months) compared to the group receiving the gp100 peptide vaccine alone (6.4 months). This suggests that the combination of the gp100 peptide vaccine with immunotherapy can enhance survival outcomes for patients with metastatic melanoma [150]. Similarly, combining the HPV16-specific vaccine ISA101 with the anti-PD-1 antibody nivolumab demonstrated promising efficacy in a phase 2 clinical trial (NCT02426892) for patients with incurable HPV-16-positive cancer in 2019 [161]. The study enrolled 24 patients and reported an overall response rate of 33%, with a median OS of 17.5 months, suggesting enhanced tumoricidal effects compared to PD-1 inhibition alone [161]. These results indicate potential benefits of combining therapeutic vaccines with immune checkpoint inhibitors (Figure 2). The detailed experimental contents are summarized in Table 3.

### 5.2. Combination Therapy with Chemotherapy

The combination of chemotherapy and peptide-based tumor vaccines has shown promising results in enhancing anti-tumor immune responses and improving clinical outcomes for cancer patients [163]. This synergistic approach leverages the cytotoxic effects of chemotherapy to reduce tumor burden and modulate the tumor microenvironment, making it more conducive to immune activation, while peptide-based vaccines specifically target tumor-associated antigens to elicit robust immune responses [165]. Chemotherapy can increase the immunogenicity of tumors by inducing immunogenic cell death, which releases tumor antigens and danger signals that enhance the recruitment and activation of APCs [165]. This process is crucial for the efficacy of peptide-based vaccines, as it amplifies the presentation of tumor antigens in the context of major histocompatibility complex (MHC) molecules, thereby improving the activation of CTLs [166]. Tumors often create an immunosuppressive microenvironment that hinders effective immune responses. Chemotherapeutic agents such as cyclophosphamide and paclitaxel can deplete Tregs and MDSCs, which are key players in immune suppression within the tumor microenvironment [167]. By reducing the population of these immunosuppressive cells, chemotherapy enhances the efficacy of peptide-based vaccines, allowing for more robust activation and proliferation of effector T cells [168] (Figure 2).

Several studies have investigated the efficacy of combining peptide tumor vaccines with chemotherapy, yielding mixed results. In 2011, Slingluff et al. conducted a multicenter randomized trial to evaluate the effects of melanoma-associated helper peptides and cyclophosphamide (CY) on the immunogenicity of a multipeptide melanoma vaccine [155]. Involving 167 patients with resected stage IIB to IV melanoma, the trial revealed that melanoma-associated helper peptides paradoxically decreased CD8(+) T-cell responses, while CY pretreatment had no significant immunologic or clinical effect [155]. Similarly, in 2014, the TeloVac trial by Middleton et al. assessed the efficacy of the telomerase peptide vaccine GV1001 combined with gemcitabine and capecitabine in 1062 patients with locally advanced or metastatic pancreatic cancer [141]. This study found that adding GV1001 to chemotherapy did not significantly improve OS compared to chemotherapy alone, highlighting the need for new strategies to enhance immune responses during chemotherapy [141]. In another study, Weller et al. evaluated the combination of rindopepimut and temozolomide in a randomized, double-blind, international phase 3 trial involving 745 patients with newly diagnosed, EGFRvIII-expressing glioblastoma [140]. The trial showed no significant difference in OS between the treatment groups, indicating that rindopepimut did not increase survival [140]. Conversely, in 2017, Hijikata et al. conducted a phase I trial on RNF43 peptide-related immune cell therapy combined with low-dose cyclophosphamide in patients with advanced solid tumors, showing potential clinical efficacy with stable disease in six out of ten patients and an increase in tumor-reactive CD8+ T cells [162]. Additionally, in 2017, Shirahama et al. reported positive outcomes in a randomized phase II trial of personalized peptide vaccination (PPV) combined with low-dose cyclophosphamide (CPA) in advanced biliary tract cancer patients, demonstrating significantly improved PFS and OS compared to PPV alone, suggesting the potential benefit of CPA in enhancing PPV efficacy by preventing IL-6-mediated immune suppression [163]. While these studies highlight the complexity of combining peptide tumor vaccines with chemotherapy, they also illustrate the potential of this combination therapy (Figure 2). The detailed experimental contents are summarized in Table 3.

### 5.3. Combination Therapy with Radiotherapy

Combining peptide tumor vaccines with radiation therapy leverages the complementary mechanisms of both treatments to enhance anti-tumor efficacy [48]. Radiation therapy can induce immunogenic cell death, releasing TAAs and facilitating their uptake by APCs such as dendritic cells, thereby boosting the immune system’s response [165,169]. Radiation also enhances the expression of MHC class I molecules and co-stimulatory molecules on tumor cells and APCs, improving antigen presentation to T cells [169,170]. This strategic combination enhances T-cell activation and proliferation, leading to potentially improved clinical outcomes in cancer immunotherapy [171,172] (Figure 2).

In recent years, the combination of peptide tumor vaccines with radiotherapy has shown promising results in clinical trials. In 2014, Iinuma et al. conducted a phase I clinical study combining a multiple epitope peptide vaccine with chemoradiation therapy (CRT) in patients with unresectable esophageal squamous cell carcinoma (ESCC) [156]. This study involved 11 HLA-A positive patients who received a vaccine comprising five peptides (TTK, URLC10, KOC1, VEGFR1, and VEGFR2) alongside CRT [156]. The treatment was well tolerated and induced peptide-specific cytotoxic T-lymphocyte responses in all patients, with six patients achieving complete response (CR) and four maintaining long-term CR for up to 4.6 years, suggesting potential efficacy for unresectable ESCC [156]. Similarly, in 2017, Shen et al. conducted a phase I clinical study to evaluate the efficacy and safety of PPV combined with radiotherapy in patients with advanced hepatocellular carcinoma (HCC) [164]. Nine patients with advanced HCC, including those with multiple metastases, were treated with precise radiotherapy and PPV-based cellular immune therapy, leading to significant decreases in AFP levels and partial responses in three patients, with stable disease in three others [164]. This combined therapy was well tolerated, with no significant liver, kidney, or severe hematological side effects observed, indicating its feasibility and effectiveness as a treatment strategy for advanced HCC [164]. The integration of radiotherapy and peptide-based tumor vaccines represents a promising strategy to enhance anti-tumor immunity and improve clinical outcomes, offering more effective and durable tumor control (Figure 2). The detailed experimental contents are summarized in Table 3.

## 6. Administration and Monitoring Protocols

Initially, patients undergo eligibility screening and baseline measurements to determine their suitability for treatment [11]. Vaccines are administered via subcutaneous or intradermal injections. Typically, the vaccination schedule includes an initial series of doses followed by booster injections [10,11,37]. The dosage of peptide-based cancer vaccines can vary, but a common dose is around 1 mg per injection [9], sometimes combined with low-dose CPA to reduce regulatory T cells, with a dose of 100 mg/day for seven days before vaccination [163]. Peptide-based cancer vaccines are administered subcutaneously or intradermally, often with adjuvants such as GM-CSF or IFA to enhance the immune response [9,37,173]. This approach has been used in various cancers, including prostate cancer, for which a poxviral-based PSA vaccine is administered initially every two weeks for the first month, followed by monthly doses [174].

Furthermore, mucosal administration of peptide tumor vaccines has been demonstrated to be easier to manage and more cost-effective, offering a promising approach to elicit strong and localized immune responses [175,176]. Among them, chitosan-based polyelectrolyte complexes (PECs) have been extensively applied in drug delivery due to their excellent biocompatibility, biodegradability, and bioadhesive properties, making them ideal for mucosal administration [177]. Mucosal routes of administration for peptide tumor vaccines, including oral, nasal, sublingual, and other mucosal surfaces, offer significant advantages by eliciting robust and localized immune responses [178]. Oral vaccines, often encapsulated in nanoparticles or liposomes, are absorbed through the gut-associated lymphoid tissue, inducing strong mucosal and systemic immunity [179,180,181]. Nasal vaccines target the nasal-associated lymphoid tissue, promoting rapid immune responses [182]. Sublingual vaccines, absorbed through the sublingual mucosa, bypass digestive enzymes and induce both local and systemic immunity [183]. Sublingual vaccination with the model tumor antigen ovalbumin (OVA) and the synthetic glycolipid alpha-galactosylceramide has been shown to induce both mucosal and systemic adaptive immunity [183]. These non-invasive delivery methods enhance patient compliance and improve the efficacy of peptide tumor vaccines by achieving targeted and potent immune responses, making them valuable strategies in cancer immunotherapy [177].

Monitoring involves periodic blood sampling to measure immune responses and imaging studies to track tumor progression [184]. Prognostic indicators of treatment success include immune response markers, such as T-cell activation, cytokine profiles, and antibody responses, as well as tumor response indicators, like imaging results and tumor biomarker levels [185]. Clinical outcomes are measured through OS, PFS, and quality of life [11,174,186,187].

## 7. Adverse Events: Present but Manageable

One of the most common adverse events reported in peptide-based cancer vaccines trials is injection site reactions, including redness, swelling, pain, and sometimes induration at the injection site [188]. These reactions are typically mild to moderate in intensity and resolve on their own without requiring significant medical intervention [188]. For instance, in the clinical trial of the HER2/neu peptide-based cancer vaccine (NeuVax) for breast cancer, injection site reactions were among the most frequently observed side effects, often presenting as localized redness and swelling, which generally subsided without further treatment [119]. Similarly, studies on the NY-ESO-1 peptide-based cancer vaccine in melanoma and ovarian cancer have documented similar injection site reactions, emphasizing their transient and manageable nature [121].

Meanwhile, peptide-based cancer vaccines can induce various autoimmune symptoms depending on the targeted antigen and the patient’s predisposition [186]. For instance, researchers found that patients with metastatic melanoma treated with a peptide-based cancer vaccine developed vitiligo, characterized by the loss of skin pigmentation due to the immune system attacking melanocytes [186]. In some cases, the robust immune activation induced by peptide-based cancer vaccines can lead to a systemic inflammatory response known as cytokine release syndrome (CRS) [116,121]. CRS is characterized by high fever, hypotension, and multi-organ dysfunction, requiring immediate medical attention [116,121]. For instance, a phase I trial investigated the efficacy of NY-ESO-1-specific TCR-engineered T-cell therapy combined with a lymph node-targeted nanoparticle peptide vaccine for advanced soft tissue sarcoma, treating three patients with refractory synovial sarcoma, two of whom developed CRS with low to moderately elevated cytokine levels [189]. Although adverse events still exist, peptide-based cancer vaccines still show good safety compared with traditional chemoradiotherapy [190].

## 8. Adjuvants

Immune adjuvants play a crucial role in peptide-based cancer vaccines by enhancing the body’s immune response to the administered antigens, working through mechanisms such as stimulating APCs, promoting cytokine release, and inducing strong Th1 and Th2 immune responses [191]. Adjuvants like aluminum salts (alum) create depots for slow antigen release, while others like MF59 and AS04 enhance APC recruitment and activation, and some, such as CpG oligodeoxynucleotides (ODNs) and MPL, activate TLRs to stimulate robust cellular and humoral immunity [30,192,193]. A recent study has also highlighted that certain peptides also possess adjuvant properties [194]. Specifically, a nanoliposomal vaccine containing the long multi-epitope peptide E75-AE36 combined with PADRE has been shown to induce a robust immune response in the TUBO mouse model of breast cancer [194]. This formulation elicited superior CD4+ and CD8+ T-cell responses and significantly enhanced IFN-γ production, resulting in a substantial reduction in tumor growth and an increase in lifespan compared to other formulations [194]. Meanwhile, GM-CSF enhances the immune response in peptide-based cancer vaccines by promoting dendritic cell differentiation and maturation, improving peptide antigen presentation to T cells, and stimulating pro-inflammatory cytokine secretion, with clinical studies showing that GM-CSF combined with peptide-based cancer vaccines is safe, well tolerated, and effective in increasing T-cell responses and PFS in melanoma patients [119,195,196].

### 8.1. GM-CSF

GM-CSF is an effective adjuvant for peptide-based cancer vaccines due to its ability to enhance the immune response through promoting the differentiation and maturation of DCs, which are critical for effective antigen presentation and subsequent T-cell activation [197,198]. Additionally, GM-CSF improves the presentation of peptide antigens to CD4+ and CD8+ T cells, enhances the migration of DCs to lymphoid tissues, and stimulates the secretion of pro-inflammatory cytokines, thereby generating a robust and sustained anti-tumor immune response [199,200].

The use of GM-CSF in combination with peptide-based cancer vaccines has shown promise in enhancing the immune response [119,195,196]. Clinical studies have administered GM-CSF at dosages ranging from 75 to 125 µg/m^2^ subcutaneously, with minimal severe side effects and favorable safety profiles [119,186,195,196]. In a randomized phase II trial, vaccination using four melanoma peptides administered with GM-CSF in adjuvant or pulsed on dendritic cells was evaluated for clinical and immunologic responses [186]. The study demonstrated that T-cell responses to melanoma peptides were significantly higher in the GM-CSF arm compared to the dendritic cell arm, with overall immune response favoring the GM-CSF administration [186].

In a study by Atzpodien et al., ten patients with resected stage IIA-IIIC melanoma received individualized adjuvant peptide vaccinations combined with GM-CSF [195]. After a mean of 6.5 vaccination cycles, PFS was 6 months, with five patients remaining relapse-free for 1+ to 21+ months [195]. The vaccine was well tolerated, with no severe side effects [195]. Meanwhile, in the multicenter intergroup randomized placebo-controlled phase III trial E4697, Butterfield et al. investigated the effects of adjuvant GM-CSF and a multi-epitope melanoma peptide-based cancer vaccine on patients with completely resected, high-risk stage III/IV melanoma [196]. The study enrolled 815 patients and aimed to promote melanoma-specific CD8 T-cell responses through the use of GM-CSF and the melanoma antigen peptide-based cancer vaccines [196]. While the overall recurrence-free survival and OS were not significantly improved, the trial observed immunomodulatory effects, with vaccinated patients showing increased peptide-specific CD8 T-cell responses and patients receiving GM-CSF experiencing reduced percentages of circulating myeloid and plasmacytoid dendritic cells, along with the development of anti-GM-CSF neutralizing antibodies that correlated with improved clinical outcomes [196]. These clinical studies demonstrate that peptide-based cancer vaccines combined with GM-CSF are feasible and worthy of further clinical research.

### 8.2. TLR Agonists

TLR agonists, such as CpG ODNs and imiquimod, enhance the immune response by activating dendritic cells and other antigen-presenting cells, leading to the upregulation of costimulatory molecules and pro-inflammatory cytokine secretion [201]. Clinical studies have shown that CpG ODNs, administered at dosages ranging from 0.01 to 5 mg per injection, can enhance antigen-specific T-cell responses when combined with peptide-based cancer vaccines, with side effects generally limited to mild injection site reactions and transient flu-like symptoms [202,203,204]. For instance, a study combining a melanoma peptide-based cancer vaccine with CpG 7909 demonstrated enhanced CD8+ T-cell responses and increased antigen-specific immunity, with manageable side effects [204]. Meanwhile, in a study by Thomann et al., liposomal constructs co-delivering peptide epitopes and TLR agonists were developed to induce a specific anti-tumor immune response against ErbB2 protein-expressing tumor cells [205]. The researchers found that liposomes containing TLR2/6 agonists were more effective than those with TLR2/1 agonists in eradicating these tumors [205]. Additionally, incorporating mannosylated ligands into the liposomes enhanced their therapeutic efficiency, allowing for treatment with lower quantities of both TLR ligands and peptide epitopes [205].

For the overview of commonly used adjuvants in clinical practice and their characteristics, refer to Table 4.

## 9. Targeted Delivery Systems

Peptide-based cancer vaccines utilize various delivery systems to enhance their stability, targeting, and immunogenicity, including liposomes, polymeric micelle, and gold nanoparticle (AuNP) [91,205,212]. Liposomes and nanoparticles have been shown in numerous preclinical studies to protect peptides from degradation and enhance efficient antigen presentation, underscoring their potential to improve the effectiveness of peptide vaccines [91,205,212,213].

### 9.1. Utilizing Ligands for Enhanced Therapeutic Efficacy

Nanoparticles functionalized with various ligands significantly enhance the targeted delivery of peptide tumor vaccines, for example, folate-conjugated AuNPs, which target cancer cells [214]. Additionally, polymeric nanoparticles like RGD-functionalized micelles target integrins on tumor vasculature [215]. Moreover, liposomes functionalized with anti-ErbB-2 antibodies can specifically target ErbB-2 positive cancer cells, thereby enhancing the delivery and efficacy of peptide vaccines [216]. A recent study demonstrated that a liposome-based cancer vaccine containing ErbB-2 peptide and ovalbumin peptide OVA323-339 generated a rapid and high-titer anti-ErbB-2 antibody response in mice, increasing specific humoral immune responses by 7.3-fold in just 7 days [216]. This targeted approach reduced viable ErbB-2 overexpressing tumor cells in vitro by 96%, highlighting the potential of this delivery strategy to induce tumor cell death [216]. These targeted delivery systems improve the specificity, efficiency, and therapeutic outcomes of peptide vaccines in cancer immunotherapy.

### 9.2. Liposomes

Composed of lipid bilayers, liposomes can encapsulate peptides within their aqueous core or incorporate them into their lipid bilayer, shielding them from degradation and enabling controlled release [217]. They facilitate efficient uptake by immune cells through endocytosis or membrane fusion, and their surface can be modified with ligands or antibodies for targeted delivery [217]. Liposomes also act as adjuvants, boosting the immunogenicity of the encapsulated peptides [218]. These systems have shown promising results in clinical applications, such as combining LNP-based vaccines with peptide-based cancer vaccines to further enhance antitumor efficacy [212,218]. In 2015, Boks et al. demonstrated that liposomes containing the melanoma-associated antigen glycoprotein 100280-288 peptide and the TLR4 ligand monophosphoryl lipid A (MPLA) significantly enhance antigen-specific T-cell responses by dermal DCs through improved antigen presentation [219]. Their study using a human skin explant model showed that MPLA-modified liposomes were efficiently taken up by CD1a(+) and CD14(+) dermal DCs, resulting in a higher induction of CD8(+) T-cell responses compared to non-modified liposomes or soluble MPLA [219]. Recently, Mohamad-Gabriel Alameh et al. demonstrated that lipid nanoparticles (LNPs) enhance the efficacy of mRNA and protein subunit vaccines by inducing robust T follicular helper cell and humoral responses [105]. Their study found that LNPs promote the induction of strong T follicular helper cell, germinal center B-cell, long-lived plasma cell, and memory B-cell responses, resulting in durable and protective antibodies in mice [105]. The success of these experiments has brought confidence to the development of liposomes’ targeted delivery systems for peptide-based cancer vaccines.

### 9.3. Polymeric Micelle

Polymeric micelle-based delivery systems are pivotal in enhancing the efficacy of peptide tumor vaccines by encapsulating hydrophobic peptides within their hydrophobic core, thereby improving stability, bioavailability, and targeted delivery [212]. These nanosized colloidal particles, formed by the self-assembly of amphiphilic block copolymers, feature a hydrophobic core and a hydrophilic shell, which protects peptides from degradation and enhances their solubility and circulation time [212,220]. Polymeric micelles, composed of biocompatible and biodegradable materials, offer improved stability, targeting, and versatility, making them a promising strategy for cancer vaccines [221]. Despite challenges in optimizing formulations, targeting efficiency, and scalability, polymeric micelles have shown significant potential in clinical applications, demonstrating enhanced delivery and immune responses in preclinical studies [221,222]. Recently, a well-defined, self-assembling mannosylated polymer has been developed for anticancer peptide antigen delivery, enhancing stability and cellular uptake [222]. This polymer–peptide conjugate forms sub-100 nm micelles at physiological pH, improves dendritic cell activation, and enhances antigen-specific T-cell responses, leading to higher antitumor immunity in tumor-bearing mice compared to a free peptide antigen [222]. Meanwhile, a mannosylated polymer called Man-VIPER, designed for peptide antigen delivery with endosomal release properties, has demonstrated superior efficacy in generating antigen-specific cytotoxic T cells and enhanced antitumor immunity in vivo, highlighting its potential as a powerful platform for cancer immunotherapy [223]. Ongoing research and development in this field will significantly advance cancer treatment and the creation of effective peptide vaccines.

### 9.4. AuNP

AuNP, which range from 5 to 100 nm, have shown significant potential in peptide vaccines due to their inertness in biological environments and their ability to be precisely controlled and modified for specific uses [224]. AuNPs can be readily taken up by APCs, leading to robust CTL responses [225,226]. Specifically, a recent study demonstrated that AuNPs affected APCs differently in their responses to subsequent stimulations, including the altered secretion of cytokines and chemokines and enhanced antigen presentation by DCs, leading to stronger Th1, Th2, and Th17 responses [225]. Clinical applications have shown promising results, with AuNP-conjugated peptides inducing robust immune responses [224]. AuNPs have shown promise in cancer immunotherapy by effectively delivering peptide vaccines and enhancing antigen-specific immune responses [227]. In vivo studies demonstrated that AuNP delivery of the OVA peptide, with or without the CpG adjuvant, induced strong anti-tumor activity and prolonged survival in both prophylactic and therapeutic tumor models, highlighting their potential as effective peptide vaccine carriers [227].

## 10. Conclusions

Peptide-based cancer vaccines represent a promising approach in cancer immunotherapy. When combined with other immunotherapies, targeted therapies, and chemoradiotherapy, these vaccines hold significant potential to revolutionize cancer treatment. However, the absence of FDA-approved peptide-based cancer vaccines highlights the need to overcome challenges such as tumor immune escape and loss of antigen expression. To enhance efficacy, it is crucial to identify and optimize highly immunogenic epitopes and develop appropriate adjuvants and targeted delivery systems. These strategies are poised to improve patient outcomes and offer more effective and personalized cancer therapies.

## Figures and Tables

**Table 1:** Summary of characteristics of different types of tumor antigens.

Target Antigens	Class of Tumor Antigen	Description	Tumor Specificity	Example of Tumor Antigen	Immunogenicity	Immunotherapy Strategy	References
TAAs	Overexpressed antigens	Normal proteins overexpressed in tumor cells.	Low	HER2 in breast cancer	Moderate	Targeted antibody therapies	[70,71]
	Differentiation antigens	Proteins associated with the differentiated state, present in both tumor and tissue of origin.	Low	MART-1 in melanoma	High	Peptide vaccines, T-cell therapies	[72,73]
	Cancer-testis antigens	Normally expressed only in the testis but found in various tumors.	Moderate	NY-ESO-1 in melanoma	High	Cancer vaccines, adoptive T-cell transfer	[74,75,76]
	Oncofetal antigens	Expressed during fetal development and re-expressed in tumors.	Low	CEA in colorectal cancer	Moderate	Antibody–drug conjugates, vaccines	[77,78]
TSAs	Viral antigens	From oncogenic viruses, absent in normal cells.	High	HPV E6 and E7 in cervical cancer	High	Preventive vaccines, therapeutic vaccines	[79,80,81]
	Neoantigens	Unique antigens from tumor-specific alterations.	High	KRAS G12D in various cancers	Very High	Personalized neoantigen vaccines, TCR-engineered T cells	[82,83,84]
	Mutated antigens	Arise from mutations, creating novel peptides absent in normal cells.	High	IDH1 R132H in glioma	High	Neoantigen vaccines, TCR-engineered T cells	[82,83,85,86]

TAAs: tumor-associated antigens; TSAs: tumor-specific antigens; HER2: human epidermal growth factor receptor 2; MART-1: melanoma antigen recognized by T cells 1; NY-ESO-1: New York esophageal squamous cell carcinoma 1; CEA: carcinoembryonic antigen; IDH1 R132H: isocitrate dehydrogenase 1 (with the R132H mutation); HPV E6 and E7: human papillomavirus early proteins 6 and 7; KRAS G12D: Kirsten rat sarcoma viral oncogene homolog (with the G12D mutation).

**Table 2:** Summary of the characteristics of peptide-based cancer vaccines in chronological order.

Vaccine	Mechanism	Applications	Advantages	Disadvantages	Development Time	References
NY-ESO-1 Peptide Vaccine	Targets cancer-testis antigen NY-ESO-1.	Melanoma, ovarian cancer, sarcoma	Targets a tumor-specific antigen, potential for broad application, immunogenic	Limited to NY-ESO-1-positive tumors, limited clinical data, ongoing clinical trials	Late 1990s	[120,121,127,128]
MUC1 Peptide Vaccine	Targets MUC1 protein, which is overexpressed in adenocarcinomas.	Breast cancer, pancreatic cancer	Targets a common cancer antigen, potential for broad application, immunogenic	Limited efficacy as monotherapy, high cost, ongoing clinical trials	Early 2000s	[129,130,131]
WT1 Peptide Vaccine	Targets WT1 protein to stimulate immune response.	AML, mesothelioma	Targets a universal cancer marker, potential broad application, immunogenic	Limited clinical data, ongoing clinical trials, variability in patient response	Early 2000s	[123,132,133]
NeuVax (E75)	Targets HER2/neu-derived peptides to prevent recurrence.	Breast cancer	Potential to prevent recurrence, targets specific antigen, combinable with other therapies	Limited to HER2-positive cancers, variability in patient response, ongoing clinical trials	Early 2000s	[118,134]
HER-2/neu Peptide Vaccine (GP2)	Targets HER2/neu protein, common in various cancers.	Breast cancer	Targets specific cancer marker, potential to prevent recurrence, combinable with other therapies	Limited to HER2-positive cancers, variability in patient response, ongoing clinical trials	Early 2000s	[118,135,136]
MAGE-A3 Peptide Vaccine	Targets MAGE-A3 antigen.	Melanoma	Targets specific cancer marker, potential for broad application, immunogenic	Limited to MAGE-A3-positive tumors, limited clinical data, ongoing clinical trials	Early 2000s	[122,137,138]
Rindopepimut (CDX-110)	Targets EGFRvIII mutation.	Glioblastoma	Specific to tumor mutation, potential for personalized treatment, combinable with other therapies	Limited to EGFRvIII-positive tumors, limited clinical data, high cost	Early 2000s	[139,140]
IMA901	Multipeptide vaccine targeting multiple tumor-associated antigens.	Renal cell carcinoma	Targets multiple antigens, can be personalized, potential synergy with other therapies	Limited efficacy in monotherapy, not widely available, clinical trials ongoing	Mid 2000s	[116,117]
GV1001	Targets telomerase to provoke an immune response.	Pancreatic cancer, NSCLC	Targets a universal cancer marker, potential broad application, immunogenic	Limited efficacy as monotherapy, high cost, side effects	Late 2000s	[115,141]
IDH1 Peptide Vaccine	Targets mutant IDH1 protein.	Glioma	Specific to tumor mutation, potential for personalized treatment, immunogenic	Limited to IDH1-mutant tumors, limited clinical data, ongoing clinical trials	Late 2000s	[85,142]
SurVaxM	Targets survivin to inhibit apoptosis and stimulate an immune response.	Glioblastoma	Targets a universal cancer marker, potential broad application, immunogenic	Limited clinical data, ongoing clinical trials, high cost	Early 2010s	[143,144]
KRAS Peptide Vaccine	Targets mutant KRAS protein.	Colorectal cancer, pancreatic cancer, NSCLC	Specific to tumor mutation, potential for personalized treatment, immunogenic	Limited to KRAS-mutant tumors, limited clinical data, ongoing clinical trials	Late 2010s	[124,145,146,147]

AML: acute myeloid leukemia; EGFRvIII: epidermal growth factor receptor variant III; HER2/neu: human epidermal growth factor receptor 2; IDH1: isocitrate dehydrogenase 1; KRAS: Kirsten rat sarcoma viral oncogene homolog; MAGE-A3: melanoma antigen gene-A3; MUC1: mucin 1; NSCLC: non-small-cell lung cancer; NY-ESO-1: New York esophageal squamous cell carcinoma 1; WT1: Wilms tumor 1.

**Table 3:** Summary of clinical trials of peptide tumor vaccines combined with targeted therapy, immunotherapy, and chemoradiotherapy in recent years.

Types of Combination Therapy	Condition or Disease	Combination Therapy Drugs	Peptide-Based Tumor Vaccines	Phase	Enrollment	Treatment Effect	Monotherapy	Combined Therapy	ClinicalTrials.gov ID	Reference
Peptide-based tumor vaccines and targeted therapy	Advanced or metastatic renal cell carcinoma	Sunitinib	IMA901	III	1171 patients	No improvement in OS (*p* = 0.087)	Sunitinib monotherapy group Median OS (33.67 months)	Sunitinib plus IMA901 group Median OS (33.17 months)	NCT01265901	[117]
Peptide-based tumor vaccines and immunotherapy	Metastatic melanoma	Ipilimumab	gp100	III	676 patients	Improve OS (*p* < 0.001)	gp100 monotherapy group Median OS (6.4 months months)	ipilimumab plus gp100 Median OS (10.0 months)	NCT00094653	[150]
	Human papillomavirus 16-related cancer	Nivolumab	ISA101	II	24 patients	Enhanced tumoricidal effects	33% response rate and 17.5-month median OS versus PD-1 alone		NCT02426892	[161]
Peptide-based tumor vaccines and chemotherapy	Locally advanced or metastatic pancreatic cancer	Gemcitabine and capecitabine	GV1001	III	1062 patients	Do not significantly improve OS	Chemotherapy group (7.9 months); the sequential (6.9 months, HR 1.19); concurrent chemoimmunotherapy groups (8.4 months, HR 1.05)		NCT00425360	[141]
	EGFRvIII-expressing glioblastoma	Temozolomide	CDX-110	III	745 patients	Do not increase survival (*p* = 0.93)	temozolomide monotherapy group Median OS (20.0 months)	rindopepimut plus temozolomide Median OS (20.1 months)	NCT01480479	[140]
	Advanced solid tumors	Cyclophosphamide	RNF43	I	10 patients	Stable disease in six out of ten patients and an increase in tumor-reactive CD8+ T cells	–	–	–	[162]
	Biliary tract cancer	Cyclophosphamide	Personalized peptide vaccination	II	49 patients	Significantly improved PFS (*p* = 0.008) and OS (*p* = 0.004)	personalized peptide vaccination monotherapy group (PFS median time: 2.9 months; OS median time: 5.9 months)	Low dose cyclophosphamide plus personalized peptide vaccination (PFS median time: 6.1 months; OS median time: 12.1 months)	–	[163]
Peptide-based tumor vaccines and radiotherapy	Esophageal cancer	Cisplatin and 5-fluorouracil	TTK, URLC10, KOC1, VEGFR1, and VEGFR2	I	11 patients	Well tolerated, induced peptide-specific cytotoxic T-lymphocyte responses in all patients	–	–	NCT00632333	[156]
	Advanced HCC	–	personalized peptide vaccination	I	9 patients	Well tolerated, parts of patients’ AFP levels decreased and their conditions stabilized	–	–	–	[164]

HCC: hepatocellular carcinoma; AFP: alpha-fetoprotein; OS: overall survival; PFS: progression-free survival.

**Table 4:** Common clinical adjuvants and their characteristics.

Adjuvant	Description	Mechanism	Applications	References
Alum	One of the oldest and most widely used adjuvants, including aluminum hydroxide and aluminum phosphate.	Forms a depot, enhances APC uptake, induces Th2 response.	Hepatitis A and B, DTP vaccines.	[206]
MF59	An oil-in-water emulsion containing squalene.	Enhances APC recruitment and activation, balanced Th1/Th2 response.	Influenza vaccines (e.g., Fluad).	[207]
AS04	A combination of aluminum hydroxide and monophosphoryl lipid A.	Stimulates TLR4, activates APCs, strong Th1 response.	HPV vaccine (Cervarix).	[208]
CpG ODNs	Synthetic oligodeoxynucleotides containing unmethylated CpG motifs that mimic bacterial DNA.	Activates TLR9, cytokine production, strong Th1 response.	Cancer and infectious disease vaccines.	[209]
MPLA	A detoxified derivative of LPS that retains immunostimulatory properties without toxicity.	Activates TLR4, enhances humoral and cell-mediated immunity.	HPV vaccines (AS04 system).	[193]
Saponins (e.g., QS-21)	Plant-derived compounds that form complexes with cholesterol to enhance immune responses.	Enhances immune responses, stimulates Th1/Th2.	Shingrix vaccine.	[210]
Toll-like receptor agonists	Compounds that mimic PAMPs to stimulate TLRs.	Activates TLRs, enhances innate and adaptive responses.	Cancer and infectious disease vaccines.	[211]
GM-CSF	Granulocyte-macrophage colony-stimulating factor.	Stimulates the production and activation of dendritic cells and macrophages.	Melanoma vaccines, various cancer immunotherapies.	[186]

HPV: human papillomavirus; PAMPs: pathogen-associated molecular patterns; ODNs: oligodeoxynucleotides; MPLA: monophosphoryl lipid A; LPS: lipopolysaccharide; TLR: Toll-like receptor; GM-CSF: granulocyte-macrophage colony-stimulating factor.

## Data Availability

The original contributions presented in the study are included in the article. Further inquiries can be directed to the corresponding authors.

## Glossary

AFP	Alpha-fetoprotein
AML	Acute myeloid leukemia
APCs	Antigen-presenting cells
AuNP	Gold nanoparticle
CEA	Carcinoembryonic antigen
CPA	Cyclophosphamide
CRT	Chemoradiation therapy
CRS	Cytokine release syndrome
CTLA-4	Cytotoxic T-lymphocyte-associated protein 4
CTLs	Cytotoxic T lymphocytes
DCs	Dendritic cells
EGFRvIII	Epidermal growth factor receptor variant III
ESCC	Esophageal squamous cell carcinoma
FDA	Food and Drug Administration
GM-CSF	Granulocyte-macrophage colony-stimulating factor
HER2	Human epidermal growth factor receptor 2
HCC	Hepatocellular carcinoma
HLA	Human leukocyte antigen
HPV	Human papillomavirus
IFA	Incomplete Freund’s adjuvant
IFN	Interferon
IL	Interleukin
IDH1	Isocitrate dehydrogenase 1
KRAS	Kirsten rat sarcoma viral oncogene homolog
LNPs	Lipid nanoparticles
LPS	Lipopolysaccharide
MAGE	Melanoma antigen gene
MDSCs	Myeloid-derived suppressor cells
MHC	Major histocompatibility complex
MPLA	Monophosphoryl lipid A
NSCLC	Non-small-cell lung cancer
NY-ESO-1	New York esophageal squamous cell carcinoma 1
ODNs	Oligodeoxynucleotides
OS	Overall survival
OVA	Ovalbumin
PFS	Progression-free survival
PD-1	Programmed cell death protein 1
PD-L1	Programmed death-ligand 1
PPV	Personalized peptide vaccination
PSA	Prostate-specific antigen
TAAs	Tumor-associated antigens
TCR	T-cell receptors
TLR	Toll-like receptor
Tregs	Regulatory T cells
